# Benchmarking Emergency Physician EHR Time per Encounter Based on Patient and Clinical Factors

**DOI:** 10.1001/jamanetworkopen.2024.27389

**Published:** 2024-08-13

**Authors:** Mark S. Iscoe, Arjun K. Venkatesh, Margaret L. Holland, Harlan M. Krumholz, Karen Dorsey Sheares, Edward R. Melnick

**Affiliations:** 1 Department of Emergency Medicine, Yale School of Medicine, New Haven, Connecticut; 2 Department of Biomedical Informatics and Data Science, Yale School of Medicine, New Haven, Connecticut; 3 Center for Outcomes Research and Evaluation, Yale School of Medicine, New Haven, Connecticut; 4 Department of Population Health and Leadership, University of New Haven, West Haven, Connecticut; 5 Section of Cardiovascular Medicine, Yale School of Medicine, New Haven, Connecticut; 6 Department of Pediatrics, Yale School of Medicine, New Haven, Connecticut

## Abstract

This cross-sectional study assesses the associations between patient and clinical factors and variations in time emergency department physicians spend using electronic health record (EHR) systems.

## Introduction

Electronic health records (EHRs) occupy a substantial proportion of emergency physicians’ clinical time.^1^ In ambulatory care, increased EHR time has been associated with improved care quality^2^ but also increased burnout.^3^ As emergency department (ED) crowding and acuity increase,^4^ expectations for care efficiency and complexity are anticipated to increase. We hypothesized that ED physicians’ EHR time varies based on patient and clinical factors, motivating us to benchmark encounter-level factors associated with ED physicians’ EHR use.

## Methods

This cross-sectional study of attending emergency physicians’ EHR use during adult patient care was conducted from March to August 2022 in 3 EDs at Yale New Haven Hospital system using the Epic (Epic Systems) EHR. Per the Common Rule, this study was exempt from review and informed consent because it was secondary research using identifiable data for research purposes only. We followed the STROBE reporting guideline.

Patient information and attending physicians’ EHR actions measured by the EHR audit log were extracted from the institutional data warehouse. Visits were excluded for patients seen by an attending physician only in triage; for patients with multiple visits, only the first was included. If a patient’s care was handed off between physicians, the physicians’ total EHR time was summed and attributed to the initial physician for clustering. Some EHR work completed through a track-board view (ie, a digital dashboard of active patients) is not linked to specific encounters in the audit log and was unavailable for encounter-level analysis.

Primary outcome was total attending physician EHR time per encounter. Secondary outcome was distribution of EHR time across major activities. We estimated associations between EHR time, patient characteristics, and clinical factors using multivariable linear regression with SEs clustered by initial physician. Wald tests were used to assess significance for categorical variables with more than 2 categories. Two-sided *P* < .05 was significant. Analyses were performed using Stata, version 17.0 (StataCorp).

## Results

Of 69 698 identified encounters, 35 187 encounters (for 18 953 females [53.9%] and 16 234 males [46.1%]; mean [SD] age, 54.7 [20.6] years) met the inclusion criteria for analysis. Care was provided by 79 physicians, with a median (IQR) of 428 (202-623) encounters per physician (Table).

**Table.** Patient and Clinical Characteristics and Linear Regression

Factor	Encounters, No. (%) (N = 35 187)	Linear regression	
		β (95% CI), min	*P* value
Patient			
Age, Mean (SD) y	54.7 (20.6)	0.005 (0.002-0.008)	.001
Sex			
Female	18 953 (53.9)	0 [Reference]	.30
Male	16 234 (46.1)	−0.04 (−0.12 to 0.04)	
Self-reported race and ethnicity from EHR database^a^			
American Indian or Alaska Native	58 (0.2)	−0.28 (−0.95 to 0.39)	.35
Asian	660 (1.9)	−0.07 (−0.31 to 0.17)	
Black or African American	7886 (22.4)	−0.08 (−0.17 to 0.02)	
Hispanic, Latina, Latino, or Latinx	5934 (16.9)	−0.07 (−0.20 to 0.05)	
Multiracial	205 (0.6)	−0.05 (−0.40 to 0.30)	
Native Hawaiian or Other Pacific Islander	36 (0.1)	−0.49 (−1.51 to 0.53)	
White	19.538 (55.5)	0 [Reference]	
Other or not listed^b^	870 (2.5)	−0.07 (−0.30 to 0.16)	
Emergency Severity Index^c^			
1	315 (0.9)	2.83 (1.89 to 3.76)	<.001
2	12 418 (35.3)	1.37 (0.88 to 1.85)	
3	18 850 (53.6)	1.10 (0.64 to 1.57)	
4	3166 (9.0)	0.33 (−0.21 to 0.68)	
5	438 (1.2)	0 [Reference]	
Disposition			
Admission	9967 (28.3)	0.52 (0.37 to 0.67)	<.001
Departure against medical advice	358 (1.0)	1.85 (0.92 to 2.77)	
Discharge	22 023 (62.6)	0 [Reference]	
ED observation	2371 (6.7)	0.49 (0.23 to 0.76)	
Other^b^	468 (1.3)	0.60 (0.17 to 1.03)	
Chief concern			
Abdominal pain	3552 (10.1)	0.21 (−0.11 to 0.53)	<.001
Alcohol intoxication	592 (1.7)	0 [Reference]	
Back pain	647 (1.8)	0.83 (0.44 to 1.23)	
Chest pain	2618 (7.4)	0.42 (0.10 to 0.74)	
Dizziness	886 (2.5)	0.76 (0.42 to 1.09)	
Fall	1384 (3.9)	0.79 (0.44 to 1.14)	
Motor vehicle crash	800 (2.3)	0.83 (0.43 to 1.23)	
Shortness of breath	1859 (5.3)	0.69 (0.39 to 1.00)	
Other^b^	22 849 (64.9)	0.43 (0.13 to 0.73)	
Clinical			
Resident or APP assigned	19 623 (55.8)	0.13 (0.07 to 0.33)	.21
Handoff between ED attending physicians	13 656 (38.8)	2.05 (1.92 to 2.17)	<.001
Practice setting			
Academic ED	16 364 (46.5)	0 [Reference]	<.001
Community ED	12 076 (34.3)	1.31 (0.71 to 1.91)	
Freestanding ED	6747 (19.2)	1.08 (0.62 to 1.54)	

Abbreviations: APP, advanced practice professionals; ED, emergency department; EHR, electronic health record.

^a^ Race and ethnicity data were extracted from the EHR database based on a self-reported response to a single combined prompt.

^b^ The “other” designation was selected by patients or registration staff when other categories did not apply.

^c^ The Emergency Severity Index ranged from 1 (highest acuity) to 5 (lowest acuity).

Physicians spent a median (IQR) 6.82 (3.70-11.25) minutes on the EHR per encounter. Time was spent primarily on documentation (median [IQR], 3.72 [2.15-5.88] minutes); medical record review (median [IQR], 1.05 [0.28-2.45] minutes), and order entry (median [IQR], 0.60 [0.00-1.97] minutes) (Figure).

**Figure.**
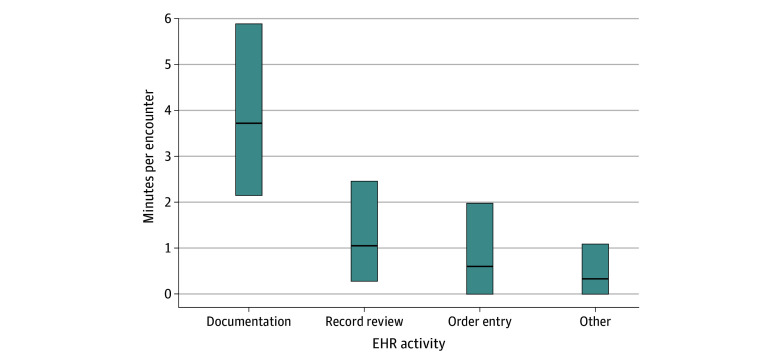
Attending Physicians’ Time Spent on EHR Use per Patient Encounter The upper and lower bounds of the box represent the 25th and 75th percentiles, respectively, with the middle line representing the median. Because this study assessed only attending physician electronic health record (EHR) use and some EHR actions were performed by resident physicians or advanced practice professionals (physician assistants, nurse practitioners, and advanced practice registered nurses), the 25th percentiles can equal 0. Other includes dispositioning (admission or discharge actions), responding to practice advisory alerts, accessing clinical care pathways, and other activities not falling under the other listed categories. Our previous study of these physicians found that they spent a median 60 minutes per 8-hour shift on the EHR track board while seeing a median 21 patients, equating to 3 additional minutes using the EHR per encounter; however, this work was likely not evenly distributed across encounters and involved workflow or team management not specific to a single patient.^1^

After adjustment, these factors were associated with encounter-level EHR time: patient age (β, 0.005 [95% CI, 0.002-0.008] minutes), Emergency Severity Index ([ESI] highest acuity encounter: β, 2.83 [95% CI, 1.89-3.76] minutes), ED disposition (admission: β, 0.52 [95% CI, 0.37-0.67] minutes), chief concern (back pain: β, 0.83 [95% CI, 0.44-1.23] minutes), practice setting (community: β, 1.31 [95% CI, 0.71-1.91] minutes), and handoff between physicians (β, 2.05 [95% CI 1.92-2.17 ] minutes) (Table).

## Discussion

We found that ED physicians spent a median of 6.82 minutes on the EHR per encounter, with more than 3 times the amount of time devoted to documentation than EHR review. Median EHR time per ED encounter was similar to time reported for outpatient encounters in procedural specialties using the Epic EHR (eg, otolaryngology^5^ and ophthalmology,^6^ 7.4 minutes and 5.9 minutes, respectively) despite the ED setting’s higher acuity. Substantial heterogeneity existed in encounter-level EHR time by ESI and presentation, however, reflective of the diversity of ED presentations. Increased EHR time associated with handoffs between physicians was found after adjustment for complexity, suggesting that handoffs entail additional EHR work. Study limitations include exclusion of track-board actions and examination of only attending physicians’ EHR use, potentially underestimating total EHR burden associated with ED care.

Given rising patient complexity^4^ and increasingly lengthy and redundant medical records, more than 1.05 minutes may be required for adequate EHR review. In addition to changes to reduce strains on ED care, new EHR tools are needed to facilitate documentation and extract and synthesize patient information, promoting safe, efficient, and high-quality care.

## Notes

Supplement.
Data Sharing Statement
