# Supplementary material for: Benchmarking Emergency Physician EHR Time per Encounter Based on Patient and Clinical Factors
Source: JAMA Netw Open. 2024 Aug 13;7(8):e2427389. doi: 10.1001/jamanetworkopen.2024.27389 (PMC11322841; doi:10.1001/jamanetworkopen.2024.27389)
Supplement: Supplement. — Data Sharing Statement [file jamanetwopen-e2427389-s001.pdf]

Data Sharing Statement

Iscoe. Benchmarking Emergency Physician EHR Time Per Encounter Based on Patient and Clinical Factors. *JAMA Netw Open*. Published August 13, 2024.
doi:10.1001/jamanetworkopen.2024.27389

Data
Data available: No

Additional Information
Explanation for why data not available: This detailed, timestamped data regarding tens of thousands of patient encounters would be difficult to truly de-identify in a way that would adequately protect both patients and clinicians.
